# Supplementary material for: Feature selection revisited in the single-cell era
Source: Genome Biol. 2021 Dec 1;22:321. doi: 10.1186/s13059-021-02544-3 (PMC8638336; doi:10.1186/s13059-021-02544-3)
Supplement: Supplementary file 2 — Additional file 2: Review history. [file 13059_2021_2544_MOESM2_ESM.docx]

Review History

**First round of review**

**Reviewer 1**

**Comments to author:**

The manuscript by Yang et al. entitled “Feature selection revisited in the single-cell era” is an article that reviews the current state of feature selection approaches that have been applied to the analysis of data from single cell cytometry and genomics technologies. In general, the review is well written and includes both a high-level description of the general approaches (filtering, wrapping and embedding) as well as short descriptions of the published variations on these approaches as they have been applied to single cell molecular data. While a summary of the field is valuable, three issues are noted:

• The authors point out that feature selection is often needed to identify the most informative feature because it may be impossible to perform an exhaustive search of the feature space to optimize downstream performance. This is indeed an important limitation. But the other limitation is that with high dimensional data, it is also common to have noisy features that correlate with the outcome variable by chance. This results in the problem of model overfitting in machine learning applications such that model performance on an independent dataset does not match performance on the training data. The authors should add a discussion of this phenomenon.

• Different downstream applications use features to perform different tasks. For example, one downstream application may want to characterize all biological pathways active in a cell of a given type or simply want to have sensitive and specific marker genes for cells of a given type. Optimal approaches for feature selection would differ depending on the specific downstream applications being addressed. The authors should also discuss this interdependency in more detail in their manuscript.

• Finally, the biggest issue with this review is that the authors do not provide any information to guide potential users as to which methods they should consider selecting based on their pros and cons and their performance. While a comprehensive evaluation that compares the performance of all relevant feature selection methods on benchmark datasets may be beyond the scope of this review, the authors should at least provide some description of the pros and cons of the different methods so that the reader has some information to guide their selection.

**Reviewer 2**

**Comments to author:**

I enjoyed reading “Feature selection revisited in the single-cell era.” The authors have done a massive amount of work to discover and organize the large number of algorithms in this space, spanning diverse feature selection strategies and diverse single cell data types. Despite the seemingly large number of algorithms in this space, single cell feature selection has received insufficient attention, perhaps because the vast majority of researchers use variants of just one algorithm, at least for feature selection in single cell RNA-seq data. Hopefully, reviews such as this will alert the field to the availability of a rich set of alternatives. This review should also help the algorithm development community realize how much is out there already, and which directions are potentially worth pursuing. Overall, I believe this will be a valuable addition to the single cell literature, and also very timely. I have provided some comments below in the spirit of trying to improve some aspects of the presentation.

Major comments

The value of this review would be greater if the authors could provide some higher-level perspective on which methods work well and which methods don't. And why, and in which circumstances. Currently, the manuscript is encyclopedic in scope and provides a lot of information, but not enough guidance. Many will read this review to figure out which algorithm they should use. If it's written purely as a catalog or a "telephone directory" listing of existing tools, they may not find what they're looking for.

Towards the end, in "upcoming domains and future opportunities," the authors provide some comments on where the field is/should be going. Broadly, the authors suggest that the future lies in more complex feature algorithms applied to more complex (multi-modal) datasets. The concern here is that complex algorithms may not be scalable. This is one general drawback - the authors have said little to nothing about scalability to a large number of cells. Since the size of single cell datasets is growing exponentially, scalability is perhaps the most important criterion for judging whether or not an algorithm is useful. Can the authors provide guidance on which algorithms are promising based on this criterion?

In general, the manuscript appears to be aimed at machine learning experts. It would be good to broaden the target audience to include non-experts. And ideally, even "lay" users who are not mathematically trained should be able to read this review and determine which method is best for their own data. If it's written only for algorithm developers with a good knowledge of machine learning, the audience will be smaller.

One possibility would be to give specific, illustrative examples. For instance, when explaining that "… ensemble feature selection methods, typically, relies on either perturbation to the dataset or hyperparameters of the feature selection algorithms …," the authors could illustrate the point by giving examples of common perturbations and widely used hyperparameters.

"While many hybrid feature selection algorithms are intuitive and numerous studies have reported favourable results compared to their individual components, a fundamental issue of these methods is their ad-hoc nature, complicating the formal analysis of their underlying properties and capabilities." Which properties and capabilities are referred to here? Again, giving concrete examples would really help the reader and it would also liven up the text. Currently, the prose is a bit dry because it relies on abstractions and generalities.

Another point regarding the above statement: at the end of the day, the only thing that matters is how well the algorithm works and how easy it is to use. The relevant properties and capabilities are: can the method scale to >1 million cells? Are the results stable (reproducibility, robustness to slight variations in the data)? Do the selected features work well in practice for the desired application (for example, classification accuracy)? Is the code easy to use and well documented? Hybrid methods are not necessarily at a disadvantage in these areas.

Minor comments

First sentence of manuscript: "… biomolecules are measured in cells and tissues with great depth and high accuracy." Single cell measurements are in fact characterized by low accuracy. This is because of the small number of molecules in a single cell and the low sensitivity of most biochemical assays. In addition, variation in sample quality, reagents and workflow introduce profound technical variation in the data.

Table 1: please provide the names of the algorithms.

The OGFSC algorithm for unsupervised feature selection (Ref. 60) is described as being distinct from the more conventional highly variable genes (HVG) approach, perhaps because the OGFSC paper makes the same claim. However, this is not true. Although the OGFSC paper states that conventional HVG-based feature selection uses the same variability threshold for all genes regardless of expression level, the reality is that HVG feature selection methods have always tuned their threshold to adjust for mean expression level. To summarize: as far as I can tell, OGFSC is merely yet another variant of HVG.

Ref. 96 cited some single cell feature selection methods not discussed in this manuscript. It may be a never-ending task to try to cite all existing single cell feature selection methods, because there are so many new ones coming up all the time. Of course, many are merely variations of the well-established HVG paradigm, with slight tweaks in how variability is quantified. Nevertheless, the methods cited in Ref. 96 should be included.

Typo: "These analyses enable us to expend the gene expression analysis …" - should be "expand" instead of "expend."

The section on feature selection methods for scATAC-seq data seems to include only supervised methods that identify differentially accessible loci. Should also mention unsupervised methods for scATAC-seq feature selection such as DUBStepR (and potentially others as well?).

In the section on feature selection for single cell imaging data, SpatialDE (Svensson et al. 2018, Nature Methods 15:343) was left out.

**Authors Response**

**Point-by-point responses to the reviewers’ comments:**

The manuscript by Yang et al. entitled “Feature selection revisited in the single-cell era” is an article that reviews the current state of feature selection approaches that have been applied to the analysis of data from single cell cytometry and genomics technologies. In general, the review is well written and includes both a high-level description of the general approaches (filtering, wrapping and embedding) as well as short descriptions of the published variations on these approaches as they have been applied to single cell molecular data. While a summary of the field is valuable, three issues are noted:

Response: We thank the reviewer for the positive review and valuable comments. We have addressed the comments and suggestions point-by-point below.

• The authors point out that feature selection is often needed to identify the most informative feature because it may be impossible to perform an exhaustive search of the feature space to optimize downstream performance. This is indeed an important limitation. But the other limitation is that with high dimensional data, it is also common to have noisy features that correlate with the outcome variable by chance. This results in the problem of model overfitting in machine learning applications such that model performance on an independent dataset does not match performance on the training data. The authors should add a discussion of this phenomenon.

*Response: We thank the reviewer for this suggestion. We have now incorporated these limitations in the revised manuscript. Specifically, we revised the introduction to include the following:*

*“The high dimensionality, redundancy, and noise commonly found in these large-scale molecular datasets create significant challenges in their analysis and can lead to a reduction in model generalisability and reliability. Feature selection, a class of computational techniques for data analytics and machine learning, is at the forefront in dealing with these challenges and has been an essential driving force in a wide range of bioinformatics applications.”*

*We have also included the following at the start of the second section “Basics of feature selection techniques”:*

*“When dealing with high-dimensional data, feature selection is an effective strategy to reduce the feature dimension and redundancy and can alleviate issues such as model overfitting in downstream analysis.”*

• Different downstream applications use features to perform different tasks. For example, one downstream application may want to characterize all biological pathways active in a cell of a given type or simply want to have sensitive and specific marker genes for cells of a given type. Optimal approaches for feature selection would differ depending on the specific downstream applications being addressed. The authors should also discuss this interdependency in more detail in their manuscript.

*Response: We agree that the choice of feature selection methods is dependent on many factors including the specific tasks at hand and downstream analyses. As suggested, we have now discussed these aspects and several other considerations for choosing feature selection methods in a new section “Applicability considerations” in the revised manuscript.*

• Finally, the biggest issue with this review is that the authors do not provide any information to guide potential users as to which methods they should consider selecting based on their pros and cons and their performance. While a comprehensive evaluation that compares the performance of all relevant feature selection methods on benchmark datasets may be beyond the scope of this review, the authors should at least provide some description of the pros and cons of the different methods so that the reader has some information to guide their selection.

*Response: We have now included discussions and recommendations to guide potential users for choosing feature selection methods in the section “Applicability considerations” of the revised manuscript. We appreciate and agree that a comprehensive evaluation of methods is beyond the scope of a review, and we note that we do not provide recommendations to a specific implementation of a method. Instead, we attempt to guide the users to a type of method (e.g., filters, wrappers, embedded and other more advanced methods) by discussing strength and weakness in terms of their applicability from various aspects such as scalability, robustness, and interpretability among others.*

Reviewer #2:

I enjoyed reading “Feature selection revisited in the single-cell era.” The authors have done a massive amount of work to discover and organize the large number of algorithms in this space, spanning diverse feature selection strategies and diverse single cell data types. Despite the seemingly large number of algorithms in this space, single cell feature selection has received insufficient attention, perhaps because the vast majority of researchers use variants of just one algorithm, at least for feature selection in single cell RNA-seq data. Hopefully, reviews such as this will alert the field to the availability of a rich set of alternatives. This review should also help the algorithm development community realize how much is out there already, and which directions are potentially worth pursuing. Overall, I believe this will be a valuable addition to the single cell literature, and also very timely. I have provided some comments below in the spirit of trying to improve some aspects of the presentation.

*Response: We appreciate the enthusiasm and constructive feedback from this reviewer. We have addressed all comments and suggestions raised by the reviewer point-by-point below.*

Major comments

The value of this review would be greater if the authors could provide some higher-level perspective on which methods work well and which methods don't. And why, and in which circumstances. Currently, the manuscript is encyclopedic in scope and provides a lot of information, but not enough guidance. Many will read this review to figure out which algorithm they should use. If it's written purely as a catalog or a "telephone directory" listing of existing tools, they may not find what they're looking for.

*Response: We thank this suggestion. We have now included a new section “Applicability considerations” in the revised manuscript, in which we provide detailed discussion of utility and applicability of feature selection methods, and practical information to guide the choice of methods for readers who are interested in the application of feature selection methods to their work. We note that, as suggested, our discussion and suggestions are at a higher-level perspective as recommendations on a specific implementation of a method requires a comprehensive benchmark study, which is beyond the scope of our review.*

Towards the end, in "upcoming domains and future opportunities," the authors provide some comments on where the field is/should be going. Broadly, the authors suggest that the future lies in more complex feature algorithms applied to more complex (multi-modal) datasets. The concern here is that complex algorithms may not be scalable. This is one general drawback - the authors have said little to nothing about scalability to a large number of cells. Since the size of single cell datasets is growing exponentially, scalability is perhaps the most important criterion for judging whether or not an algorithm is useful. Can the authors provide guidance on which algorithms are promising based on this criterion?

*Response: We agree with the reviewer that scalability is a critical criterion for assessing feature selection algorithms. We have taken this suggestion wholeheartedly and have now discussed these aspects in the new section “Applicability considerations” in our revised manuscript. In particular, we commented on the method scalability towards the feature dimension and the sample size and the reproducibility and interpretability for both classic and more advanced feature selection algorithms.*

In general, the manuscript appears to be aimed at machine learning experts. It would be good to broaden the target audience to include non-experts. And ideally, even "lay" users who are not mathematically trained should be able to read this review and determine which method is best for their own data. If it's written only for algorithm developers with a good knowledge of machine learning, the audience will be smaller. One possibility would be to give specific, illustrative examples. For instance, when explaining that "… ensemble feature selection methods, typically, relies on either perturbation to the dataset or hyperparameters of the feature selection algorithms …," the authors could illustrate the point by giving examples of common perturbations and widely used hyperparameters.

*Response: We appreciate this suggestion. We have now added additional explanations and given examples to help the readers across the manuscript. Since the goal of this review is to stimulate future research and application of feature selection methods to single-cell data, we aim to strike a balance between the machine learning experts and lay users. As mentioned in our response to the first point, we have now also included a new section “Applicability considerations”, in which we provide more practical information to guide the choice of methods for bioinformaticians and biologists who may be more interested in the application of feature selection methods. With different sections tailored for different audiences, we hope the revised manuscript will benefit both machine learning experts and non-experts.*

"While many hybrid feature selection algorithms are intuitive and numerous studies have reported favourable results compared to their individual components, a fundamental issue of these methods is their ad-hoc nature, complicating the formal analysis of their underlying properties and capabilities." Which properties and capabilities are referred to here? Again, giving concrete examples would really help the reader and it would also liven up the text. Currently, the prose is a bit dry because it relies on abstractions and generalities.

*Response: By this, we were referring to the theoretical algorithmic complexity which can provide estimations of computation time and scalability of a method. Since the hybrid methods are combinations of different feature selection methods, such theoretical analyses of algorithmic complexity can be harder to perform. We have revised the manuscript to clarify this. Nevertheless, we agree with the reviewer that this probably has very little impact on the utility and applicability of hybrid methods in practice. We hope the newly added section “Applicability considerations” further clarify these aspects.*

Another point regarding the above statement: at the end of the day, the only thing that matters is how well the algorithm works and how easy it is to use. The relevant properties and capabilities are: can the method scale to >1 million cells? Are the results stable (reproducibility, robustness to slight variations in the data)? Do the selected features work well in practice for the desired application (for example, classification accuracy)? Is the code easy to use and well documented? Hybrid methods are not necessarily at a disadvantage in these areas.

*Response: We have now discussed in detail the scalability, robustness, interpretability, and several other aspects for selecting feature selection methods and acknowledged that hybrid methods are not necessarily at a disadvantage (please see section “Applicability considerations”).*

Minor comments

First sentence of manuscript: "… biomolecules are measured in cells and tissues with great depth and high accuracy." Single cell measurements are in fact characterized by low accuracy. This is because of the small number of molecules in a single cell and the low sensitivity of most biochemical assays. In addition, variation in sample quality, reagents and workflow introduce profound technical variation in the data.

*Response: We thank the reviewer for pointing this out. We have revised the introduction accordingly. Specifically, we have included the following:*

*“While significantly higher coverage of molecules is achieved by high-throughput biotechnologies compared to traditional biochemical assays, the variation in sample quality, reagents and workflow introduce profound technical variation in the data.”*

Table 1: please provide the names of the algorithms.

*Response: Given the space limit in Table 1, we have now created a Table S1 which provides the details (including the names of the algorithms) of each method included in Table 1.*

The OGFSC algorithm for unsupervised feature selection (Ref. 60) is described as being distinct from the more conventional highly variable genes (HVG) approach, perhaps because the OGFSC paper makes the same claim. However, this is not true. Although the OGFSC paper states that conventional HVG-based feature selection uses the same variability threshold for all genes regardless of expression level, the reality is that HVG feature selection methods have always tuned their threshold to adjust for mean expression level. To summarize: as far as I can tell, OGFSC is merely yet another variant of HVG.

*Response: We appreciate the explanation and have noted this in the revised manuscript (section “Feature selection in single-cell transcriptomics”).*

Ref. 96 cited some single cell feature selection methods not discussed in this manuscript. It may be a never-ending task to try to cite all existing single cell feature selection methods, because there are so many new ones coming up all the time. Of course, many are merely variations of the well-established HVG paradigm, with slight tweaks in how variability is quantified. Nevertheless, the methods cited in Ref. 96 should be included.

*Response: We thank the reviewer for this suggestion. Given that the methods cited in ref. 96 primarily focus on scRNA-seq data clustering and use similar variability measures for gene filtering, we think it should be sufficient to summarise such kind of application and point the readers to ref. 96 for detailed comparison of each of these variants. We have revised the manuscript to clarify these (section “Feature selection in single-cell transcriptomics”, second paragraph).*

Typo: "These analyses enable us to expend the gene expression analysis …" - should be "expand" instead of "expend."

*Response: We have corrected this typo.*

The section on feature selection methods for scATAC-seq data seems to include only supervised methods that identify differentially accessible loci. Should also mention unsupervised methods for scATAC-seq feature selection such as DUBStepR (and potentially others as well?).

*Response: We note that DUBStepR was designed for scRNA-seq data and therefore was included in the “Feature selection in single-cell transcriptomics” section of our original submission. Nevertheless, we acknowledge that the authors of DUBStepR mentioned that it is a general approach and can be applied to other data types such as scATAC-seq. We have now revised the manuscript to also include this method in the “Feature selection in single-cell epigenomics” section.*

In the section on feature selection for single cell imaging data, SpatialDE (Svensson et al. 2018, Nature Methods 15:343) was left out.

*Response: We thank the reviewer for preferring us to this paper. We have now included this reference in the section “Feature selection in single-cell imaging data” of the revised manuscript.*

**Second round of review**

**Reviewer 1**

The authors have satisfactorily responded to my previous review.

**Reviewer 2**

The revised manuscript is substantially improved and my comments have mostly been well addressed. The new “Applicability Considerations” section helps, though it’s written at a very high level of abstraction. Perhaps necessarily so, because we need more benchmarking studies before we can say something more specific. Nevertheless, it would be good to emphasize a bit more strongly that the details of how the algorithm is structured and implemented are critical to scalability and robustness. In particular, if the authors could provide the list of algorithms that have demonstrated scalability to large datasets, that would help users quite a bit. The proof of the pudding is in the eating.

I have only one other major comment: I didn’t notice this earlier, but among the large number of papers cited in Table 1, I found a few that one would not really think of as single cell feature selection methods. In fact, some of them don’t even present a new method – they merely use existing tools published elsewhere.

For example, I manually checked Refs. 61-64: 1) scATAC-pro (Ref. 61) doesn’t include a feature selection step. I couldn’t find any reference to feature selection in that paper. The tool allows calculation of differential accessibility using the standard Wilcoxon test, but I’m not sure this qualifies as a feature selection algorithm for scATAC-seq data (otherwise even the Kolmogorov-Smirnov test or a simple t-test could be defined as a single cell feature selection method). 2) Scasat (Ref. 62): same comment as above – this tool doesn’t do feature selection (the paper only refers to feature extraction). 3) SnapATAC (Ref. 63) – same comment as above. It merely uses EdgeR to identify differentially accessible regions. There’s no new feature selection algorithm here. 4) Ref. 64 – same comment as above. This paper doesn’t describe any feature selection method. They merely use the Signac tool to identify differentially accessible regions. I did not have time to manually check all of the other methods listed in Table 1, but it’s possible some of them may not belong here either. There are many algorithms for calling differentially expressed genes (“DE callers”) or differentially accessible peaks (“DA callers”). These tools have their own review papers – they are a separate category, in my view. From a purely technical machine learning perspective, one could formally classify them as supervised feature selection methods. However, the fact is that DE callers are not what people in the single cell field think of when they think of feature selection. Including them in this list could cause confusion.

My suggestion: include only the feature selection algorithms that were explicitly designed for single cell clustering, classification, etc. If the authors feel it’s important to include single cell DE callers and DA callers as well, perhaps they could go in a separate table and a separate section of the text. Along similar lines: it would be extremely useful to create separate lists for supervised and unsupervised feature selection methods, because they have different applications (rather than putting them all in one list as though they are interchangeable).

Minor comment Ref. 90 is a biorxiv preprint – would be better to cite the journal version.

**Authors Response**

**Point-by-point responses to the reviewers’ comments:**

Reviewer #2:

The revised manuscript is substantially improved and my comments have mostly been well addressed.

The new "Applicability Considerations" section helps, though it's written at a very high level of abstraction. Perhaps necessarily so, because we need more benchmarking studies before we can say something more specific. Nevertheless, it would be good to emphasize a bit more strongly that the details of how the algorithm is structured and implemented are critical to scalability and robustness. In particular, if the authors could provide the list of algorithms that have demonstrated scalability to large datasets, that would help users quite a bit. The proof of the pudding is in the eating.

*Response: As suggested, we have now emphasized more on the scalability and robustness of the algorithm. In particular, we added the following at the start of the section “Applicability considerations”:*

*“In practice, scalability and robustness are critical in choosing feature selection techniques and are largely dependent on the algorithm structure and implementation.”*

I have only one other major comment:

I didn't notice this earlier, but among the large number of papers cited in Table 1, I found a few that one would not really think of as single cell feature selection methods. In fact, some of them don't even present a new method - they merely use existing tools published elsewhere. For example, I manually checked Refs. 61-64:

1) scATAC-pro (Ref. 61) doesn't include a feature selection step. I couldn't find any reference to feature selection in that paper. The tool allows calculation of differential accessibility using the standard Wilcoxon test, but I'm not sure this qualifies as a feature selection algorithm for scATAC-seq data (otherwise even the Kolmogorov-Smirnov test or a simple t-test could be defined as a single cell feature selection method).

2) Scasat (Ref. 62): same comment as above - this tool doesn't do feature selection (the paper only refers to feature extraction).

3) SnapATAC (Ref. 63) - same comment as above. It merely uses EdgeR to identify differentially accessible regions. There's no new feature selection algorithm here.

4) Ref. 64 - same comment as above. This paper doesn't describe any feature selection method. They merely use the Signac tool to identify differentially accessible regions.

I did not have time to manually check all of the other methods listed in Table 1, but it's possible some of them may not belong here either.

There are many algorithms for calling differentially expressed genes ("DE callers") or differentially accessible peaks ("DA callers"). These tools have their own review papers - they are a separate category, in my view. From a purely technical machine learning perspective, one could formally classify them as supervised feature selection methods. However, the fact is that DE callers are not what people in the single cell field think of when they think of feature selection. Including them in this list could cause confusion.

My suggestion: include only the feature selection algorithms that were explicitly designed for single cell clustering, classification, etc. If the authors feel it's important to include single cell DE callers and DA callers as well, perhaps they could go in a separate table and a separate section of the text.

Along similar lines: it would be extremely useful to create separate lists for supervised and unsupervised feature selection methods, because they have different applications (rather than putting them all in one list as though they are interchangeable).

*Response: We appreciate the above suggestions from the reviewer regarding differential expression (DE) and, more generally, other “differential analysis” methods. We note that DE (e.g., t-test) and other differential analysis methods are frequently used for feature selection in practice and have been traditionally classified as filter feature selection techniques as can be seen in the landmark review article “A review of feature selection techniques in bioinformatics” by Saeys et al. (PMID: 17720704).*

*Given their common usage as feature selection techniques in bioinformatics community, we believe it is useful to include DE and differential analysis methods as filters in this review. In particular, we have made sure this is introduced and clarified early on. As can be seen from our introduction of filter methods in section “Basic of feature selection techniques” in our initial submission, we have noted the following:*

*“Filter methods typically rank the features based on certain criteria that may facilitate other subsequent analyses (e.g., discriminating samples) and select those that pass a threshold judged by the filtering criteria (Fig. 1a). In bioinformatics applications, commonly used criteria are univariate methods such as t-statistics, on which most ‘differential expression’ (DE) methods for biological data analysis are built (11), and multivariate methods that takes into account relationships among features (12).”*

*While some of the tools we referred to in the review didn’t use the phrase “feature selection” in their texts, they did use DE or other differential analysis methods to filter for genes and other molecular features to reduce the dimensionality of data for their downstream analysis. Such usage constitutes an application of filter for feature selection.*

Minor comment

Ref. 90 is a biorxiv preprint - would be better to cite the journal version.

*Response: We thank the reviewer for this. We have now replaced the citation with the journal version.*
